# Identification of Senescence- and Inflammation-Related Genes and Immune Microenvironment Characterization in Intracranial Aneurysms

**DOI:** 10.2174/0118715303420035251023051847

**Published:** 2026-01-02

**Authors:** Xiaoyan Li, Yingying Li, Le Zhang, Jie Mao, Bin Li

**Affiliations:** 1 Department of Neurology, Anhui No.2 Provincial People’s Hospital, Hefei, 230041, China;; 2 Graduate School, Bengbu Medical University, Bengbu, 233000, China;; 3 Department of Neurosurgery, Longgang Central Hospital of Shenzhen, Shenzhen, 518035, China

**Keywords:** Intracranial aneurysm, senescence, inflammation, WGCNA, transcription factor-miRNA regulatory network, immune infiltration

## Abstract

**Introduction:**

Intracranial aneurysm (IA) is a cerebrovascular disease that lacks effective methods for early diagnosis and risk prediction. Considering the pivotal roles of senescence and inflammation in many diseases, this study aimed to identify related biomarkers for IA and explore their underlying mechanisms.

**Methods:**

The GSE122897 and GSE75436 datasets were obtained from the Gene Expression Omnibus (GEO) database. Senescence and inflammation scores were calculated using single-sample GSEA (ssGSEA), and weighted gene co-expression network analysis (WGCNA) was performed to identify relevant modules and hub genes. Differentially expressed genes (DEGs) were determined with the DESeq2 package, followed by conducting LASSO regression and support vector machine-recursive feature elimination (SVM-RFE) to screen key genes. Immune infiltration was analyzed using CIBERSORT and ESTIMATE algoritms, and correlations between key genes and immune cells were assessed. Finally, transcription factor (TF)-miRNA regulatory networks were constructed using the JASPAR package and ENCORI database.

**Results:**

IA samples exhibited significantly higher senescence and inflammation scores in comparison to the controls. A total of 858 hub genes identified by WGCNA were intersected with the DEGs for further refinement by LASSO and SVM-RFE, ultimately yielding *PTCH1*, *ACACB*, *DRD1*, and *SLC25A21-AS1* as the candidate genes for IA. Immune infiltration analysis showed that the expression of these genes was associated with several immune cells, including NK cells. Moreover, IA samples had higher ESTIMATEScore, ImmuneScore, and StromalScore, which were all negatively correlated with the expression of the four genes. TF-miRNA network analysis revealed 67, 71, and 76 potential TF regulators for *PTCH1, ACACB,* and *SLC25A21-AS1*, respectively.

**Discussion:**

These findings indicated that senescence and inflammation contributed to IA pathogenesis and may regulate disease progression by modulating the immune microenvironment, highlighting their dual role in IA.

**Conclusion:**

*PTCH1*, *ACACB*, and *SLC25A21-AS1* were identified as potential biomarkers associated with senescence and inflammation in IA, providing novel insights into its molecular mechanisms and potential diagnostic or therapeutic targets.

## 1 INTRODUCTION

Intracranial aneurysm (IA) is a complex, multifactorial cerebrovascular disorder affecting approximately 3% of the general population [1, 2]. The mortality rate of aneurysmal subarachnoid hemorrhage following IA rupture has been reported to reach 30% [3]. Despite available treatment options, including microsurgical and endovascular therapies, most patients fail to receive timely treatment due to the asymptomatic nature of cerebral aneurysms and the high risk associated with surgical procedures [4]. Currently, as the risk of IA rupture remains unpredictable, clinicians have to decide on conservative or preventive treatment for IA patients based on CT or MRI diagnosis [5]. Hence, effective biomarkers should be discovered to improve early detection of IA and prediction of IA rupture.

A variety of damage-inducing stimuli, including senescence, DNA damage, oncogene activation or inactivation, and inflammatory cytokines, can induce senescence [6, 7]. Previous studies have reported cellular senescence as an endogenous tumor-suppressive mechanism that inhibits cancer development through permanently limiting the growth and malignant transformation of damaged cells [8]. Previous studies have demonstrated that oncogene-induced senescence inhibits the growth of malignancies, such as pancreatic ductal adenocarcinoma [9] and T-cell lymphoma [10], in transgenic mice. Senescence cells maintain metabolic activity and are capable of secreting pro-inflammatory cytokines and chemokines, creating a pro-tumorigenic microenvironment to promote carcinogenesis [11, 12]. Inflammation, which is characterized by vascular responses, immune cell recruitment, and release of molecular mediators, is an adaptive response of the body to infection and tissue injury [13, 14]. Chronic inflammation has been considered a hallmark of cancer development as it promotes cell survival and proliferation, extracellular matrix remodeling and degradation, and weakens the vascular barrier [15, 16]. Although the roles of senescence- and inflammation-related genes have been extensively studied in tumor biology, their relevance in non-neoplastic vascular diseases, such as IA, has received limited research attention. Evidence indicated that both vascular aging and chronic inflammation are critically involved in IA formation and rupture [17, 18]. For instance, vascular senescence contributes to endothelial dysfunction and matrix degradation, and impairs vascular remodeling, which are commonly observed features in IA pathology [19]. Similarly, chronic inflammatory processes in IA lesions involve leukocyte infiltration, cytokine secretion, and macrophage activation, which promote vessel wall weakening and rupture risk [20, 21]. Given these mechanistic parallels, genes associated with cellular senescence and inflammation may have the potential to serve as novel diagnostic biomarkers and therapeutic targets for IA.

In this study, we obtained scRNA-seq expression profiles of IA from public databases, assessed the senescence- and inflammation-related scores of each sample in the GSE122897 training set, and performed functional enrichment analysis on the hub genes linked to both senescence and inflammation in the GSE122897 training cohort. Next, DEGs between IA and control samples were identified to select key DEGs associated with both senescence and inflammation. We also analyzed the immune infiltration in the training set samples and explored the correlation between the key genes and immune infiltration. Overall, this study identified novel genes for IA based on senescence and inflammation, offering novel insights into the therapeutic strategies and prognostic assessment for IA.

## 2 METHODOLOGY

### 2.1 Data Collection

The scRNA-seq expression profiles of 16 cases of intracranial cortical artery control samples and 44 IA tissue samples in the GSE122897 dataset were collected from the GEO database (https://www.ncbi.nlm.nih.gov/geo/) as a training set. The GSE75436 dataset, containing 15 superficial temporal artery control samples and 15 IA tissue samples, and their complete clinical information served as a test set. Cellage database (https://genomics.senescence.info/cells/) was then used to extract 949 senescence-related genes. Furthermore, the HALLMARK_INFLAMMATORY_RESPONSE dataset was downloaded from the MSigDB database (https://www.gsea-msigdb.org/gsea/msigdb/index.jsp), and 200 inflammation-correlated genes were obtained for further examination.

### 2.2 Senescence- and Inflammation-related Scores and GSEA

We computed senescence and inflammation scores for each sample in the GSE122897 training set using ssGSEA, with genes related to senescence and inflammation as the background gene set. Gene set variation analysis (GSVA) [22] was performed to identify enriched pathways linked to inflammation and cellular senescence in IA samples, with FRIDMAN_SENESCENCE_UP and HALLMARK as the background gene sets.

### 2.3 Construction of Weighted Gene Co-expression Network Analysis (WGCNA)

After eliminating genes with an average expression of lower than 0.5, we performed WGCNA [23] using the R package “WGCNA” [24] to identify gene modules and hub genes related to senescence and inflammation in the GSE122897 training set samples. The “pickSoftThreshold” R function was used to decide the optimal soft threshold power (β=5) [25], and then hierarchical cluster analysis (soft threshold = 0.85, merge height = 0.2, minimum gene number in the module = 300) was conducted to identify gene modules. The interaction between the gene modules and IA was analyzed according to the values of gene significance (GS) and module membership (MM) [26] using the “WGCNA” package, with the genes meeting the criteria of |GS| ≥ 0.4 and |MM| ≥ 0.8 identified as hub genes. Finally, module-trait relationships were visualized by the “Heatmap” package [27], and genes exhibiting the strongest positive and negative correlations with clinical traits were selected based on the results of principal component analysis (PCA).

### 2.4 Functional Enrichment Analysis

Kyoto Encyclopedia of Genes and Genomes (KEGG) and Gene Ontology (GO; in three terms) analyses were conducted on the hub genes using the DAVID database (https://david.ncifcrf.gov/). *P*<0.05 was taken as the significance threshold to select statistically significant terms [28].

### 2.5 Differential Gene Expression Analysis

Using the “DESeq2” package, gene expression differences between IA samples and control samples in the training set were compared [29]. After background correction, quantile normalization, and probe summarization of the gene expression profile data, significant DEGs were screened under the statistical significance criteria of log_2_FC|> 1 and *p*. adj < 0.01. Next, the DEGs were visualized into heatmaps and volcanic maps utilizing the “ggplot2” package [30].

### 2.6 Screening Biomarkers Using Two Machine Learning Algorithms

LASSO analysis was performed on the above intersection genes employing the “glmnet” package [31], and those with the smallest error (lambda.min = 0.0743) were considered as the most significant genes to develop a prognostic model for IA. At the same time, the SVM-RFE algorithm was subsequently applied for further feature selection using the “e1071” R package [32]. Through recursive feature elimination, features were sequentially removed until reaching the minimal model error (N=6). Finally, genes selected by both LASSO and SVM-RFE were recognized as the feature genes, and their expressions and diagnostic performance were validated in both the GSE122897 training set and the GSE75436 test set to determine the final key biomarkers for IA.

### 2.7 Immune Infiltration Analysis

Immune cell infiltration in the GSE122897 training set samples was computed by the “CIBERSORT” R package [33] based on the LM22 signature matrix, which was collected from the official website of CIBERSORT (https://cibersortx.stanford.edu/). Immune infiltration differences between IA samples and control samples (*p*<0.05) were compared by the rank-sum test. The Spearman correlation coefficient was calculated to assess the relationship between immune cell infiltration and the key genes. The tumor microenvironment (TME) of each sample in the GSE122897 training set was evaluated by the ESTIMATE algorithm using the R package “estimate” [34]. Significant differences in the above scores between IA samples and control samples were analyzed by the rank-sum test (*p*<0.05). Spearman correlation coefficient was computed to assess the relationship between the key genes and StromalScore, ImmuneScore, and ESTIMATEScore.

### 2.8 Development of TF-miRNA Regulatory Networks

To develop the TF-regulatory networks, we used the “TFBSTools” R'package [35] and the JASPAR database (https://jaspar.elixir.no/) to predict potential TFs targeting the key genes. Subsequently, potential miRNAs targeting the genes were predicted utilizing the Encori database (https://rnasysu.com/encori/) [36]. For both analyses, only interactions supported by ≥5 experimental validations were retained to ensure high-confidence predictions.

### 2.9 Statistical Analysis

The R package (version 4.0.5) was used for all statistical analyses. Statistical differences between the two groups were compared by the Wilcoxon test. Fisher’s exact test was employed for GO and KEGG enrichment analyses. *P*-value < 0.05 was set as the threshold for statistical significance throughout the study. ^ns^*p* > 0.05; *p < 0.05, **p < 0.01, ***p < 0.001, and ****p < 0.0001.

## 3 RESULTS

### 3.1 Senescence- and Inflammation-related Scores And Pathway Enrichment Analysis

**Fig. (1):**
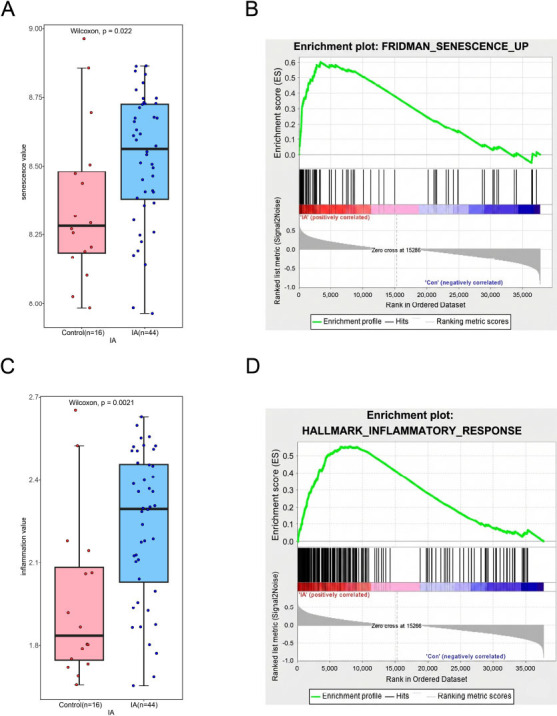
Cellular senescence and inflammation validation of IA samples in the GSE122897 training set (44 IA tissue samples and 16 intracranial cortical artery control samples). (**A**) Box line plot of the senescence gene score between IA samples and control samples. (**B**) GSEA enrichment results of senescence-related pathways. (**C**) Box line plot of inflammation gene score between IA samples and control samples. (**D**) Inflammation-related pathway GSEA results.

We calculated senescence- and inflammation-related scores for each IA sample in the GSE122897 training set. Using 949 senescence-related genes from the Cellage database as the reference gene set, we found significantly higher senescence scores in IA samples in the GSE122897 dataset in comparison to controls (*p*=0.022, Fig. **1A**). With FRIDMAN_SENESCENCE_UP as the background gene set, GSEA enrichment analysis showed significant activation of senescence-related pathways in IA patients in the GSE122897 training set (*P* = 0.0021, Fig. **1B**). For inflammation assessment, we computed inflammation scores for each sample in the GSE122897 training set, with 200 inflammation-related genes extracted from the HALLMARK_INFLAMMATORY_RESPONSE dataset serving as the background gene set. It was found that IA samples had notably higher scores of inflammation than the control samples (*p*=0.0021, Fig. **1C**). Based on the complete HALLMARK gene set, GSEA further demonstrated significant enrichment of inflammatory pathways in IA patients (Fig. **1D**). Collectively, these findings indicated dysregulation of both inflammation and cellular senescence in IA patients.

### 3.2 Identification of Hub Genes Related to Both Senescence and Inflammation in the Training Set Samples Through WGCNA

**Fig. (2):**
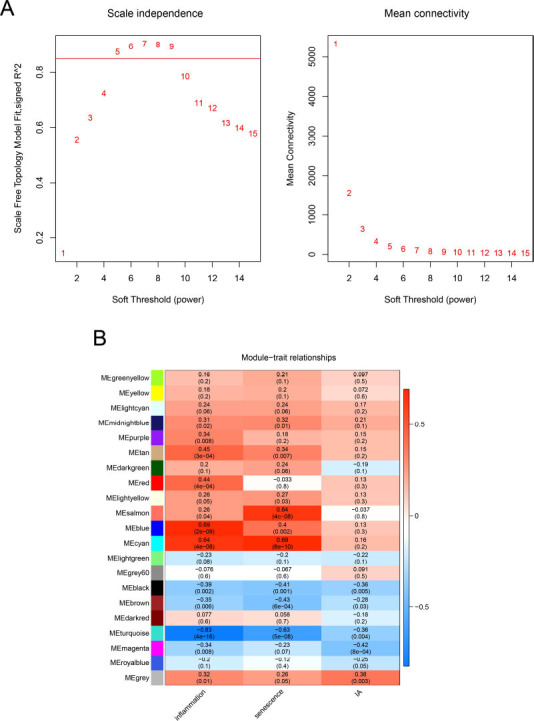
Weighted co-expression network construction with hub gene identification in the GSE122897 training cohort (44 IA tissue samples and 16 intracranial cortical artery control samples). (**A**) Soft threshold screening plot, screening the minimum value of R^2^ above 0.85 5 as the soft threshold for constructing the topological network. (**B**) Module-trait correlation diagram, in which the horizontal coordinate is the trait, the vertical coordinate is the module, the numbers in the squares are the correlation coefficients, and the numbers in the parentheses are the significance *p*-values, with the red color representing the positive correlation and the blue color representing the negative correlation.

Hub genes linked to inflammation and senescence in the training set of the GSE122897 dataset were identified employing WGCNA. To ensure a scale-free nature of the network, we selected a soft threshold power of 5 (R^2^ > 0.85, Fig. **2A**). Here, we classified 21 co-expression modules, with each module containing a minimum of 300 genes. Module-trait correlation analysis revealed that the MEcyan module was positively correlated with both inflammatory and senescence (senescence: cor = 0.69, *p* = 8e-10; inflammation: cor = 0.64, *p* = 4e-08), whereas the MEturquoise module exhibited negative correlations with senescence and inflammatory features (cor = -0.63, *p* = 5e-08; cor = -0.83, *p* = 4e-16) (Fig. **2B**). Hence, these two clinically relevant modules were used for further investigation. Subsequent analysis showed a positive association between MM and GS for both inflammation (cor = 0.69, *p*=2.6e-103) and senescence (cor = 0.66, *p*=1.3e-91) in the MEcyan module (Fig. **S1A**). Inflammation (cor = 0.89, *p*<1e-200) and senescence (cor = 0.71, *p*<1e-200) showed positive MM-GS correlation in the MEturquoise module (Fig. **S1B**). Finally, we identified 858 hub genes under the threshold of GS ≥ |0.4| and |MM| ≥ 0.8 across the two modules.

### 3.3 Functional Enrichment Analysis of Genes

**Fig. (3):**
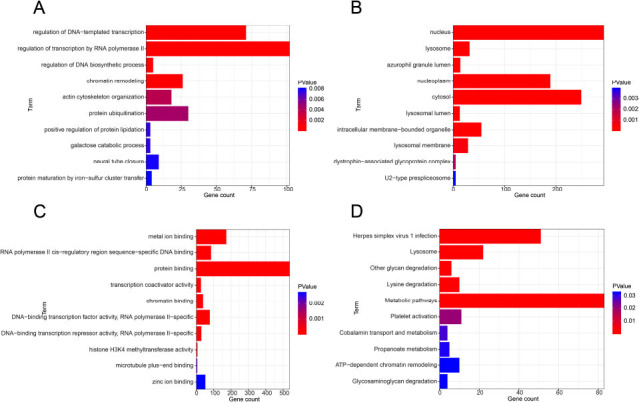
Functional enrichment analysis of hub genes. (**A**) BP bar graph in the gene GO enrichment analysis; the horizontal coordinates represent the number of genes contained within the entries, and the colors represent the significance *p*-values, increasing in significance from blue to red. (**B**) CC bar graph in the gene GO enrichment analysis. (**C**) MF bar graph in the gene GO enrichment analysis. (**D**) Bar graph of gene KEGG enrichment analysis.

We performed functional enrichment analysis on the 858 genes to investigate the regulatory role of the hub genes in the pathophysiology of IA. It was found that the hub genes were primarily enriched in DNA-templated transcription control and RNA polymerase II transcription regulation in the biological process (BP) evaluation (Fig. **3A**). Under the cellular component (CC) term, the hub genes were enriched in cytosol, intracellular membrane-bounded organelle, nucleus, and nucleoplasm (Fig. **3B**). Under the molecular function (MF) term, the hub genes were enriched in metal ion binding (Fig. **3C**). KEGG analysis demonstrated that the hub genes were particularly enriched in metabolism-related pathways, such as lysosome, metabolic pathways, and herpes simplex virus 1 infection (Fig. **3D**).

### 3.4 Screening of Differentially Expressed Genes (DEGs)

**Fig. (4):**
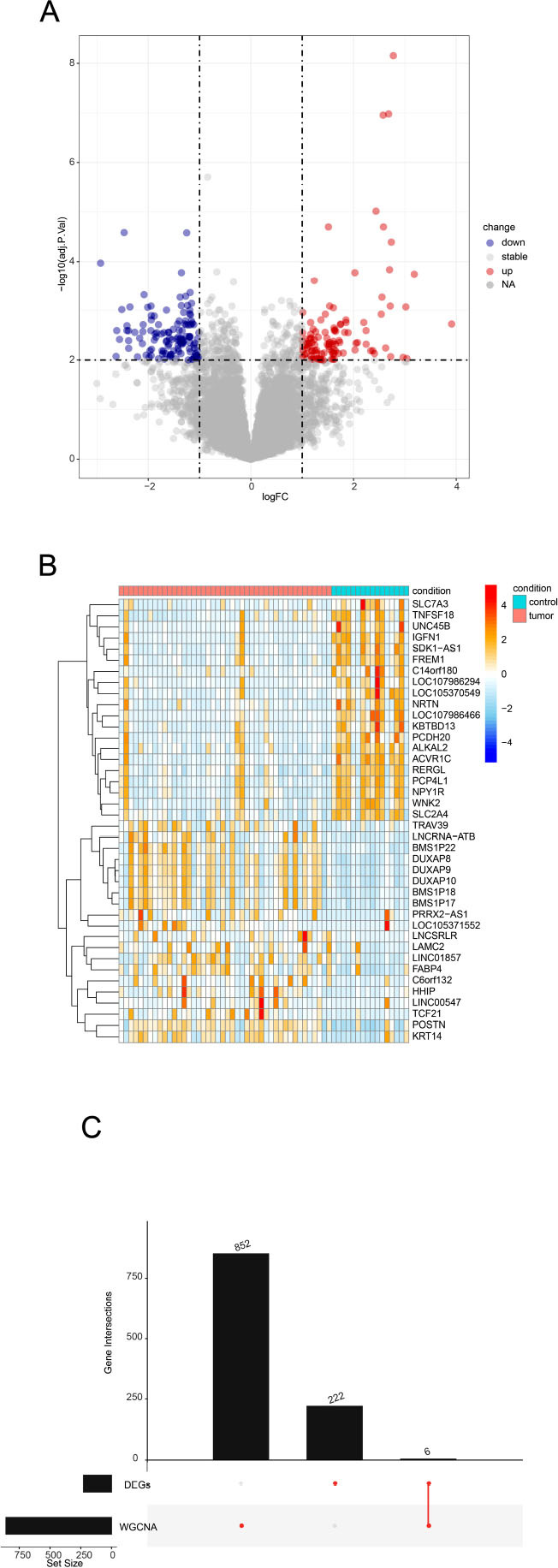
Screening of DEGs associated with senescence and inflammation in the training set samples (44 IA tissue samples and 16 intracranial cortical artery control samples). (**A**) Gene differential expression volcano plots, with horizontal coordinates representing differential expression multiplicity log_2_FC and vertical coordinates representing -log10 (*p*-value). Each point in the graph represents a gene, with red representing differentially up-regulated genes and blue representing differentially down-regulated genes. (**B**) Differential expression heatmap; red represents high expression and blue represents low expression. (**C**) Intersection upset plot of hub genes and DEGs; the left bar is the number of genes in each subset, and the top bar is the number of genes in each intersection.

In the GSE122897 training cohort, we identified a total of 228 DEGs, including 121 downregulated genes and 107 upregulated genes (Fig. **4A**). Then, the top 20 downregulated and upregulated DEGs were visualized into an expression heatmap, which displayed distinct expression patterns between the two types of samples (Fig. **4B**). Six DEGs associated with both inflammation and senescence were obtained by intersecting the DEGs and the hub genes screened by WGCNA (Fig. **4C**).

### 3.5 IA Signature Genes Screened by two Machine Learning Algorithms

**Fig. (5):**
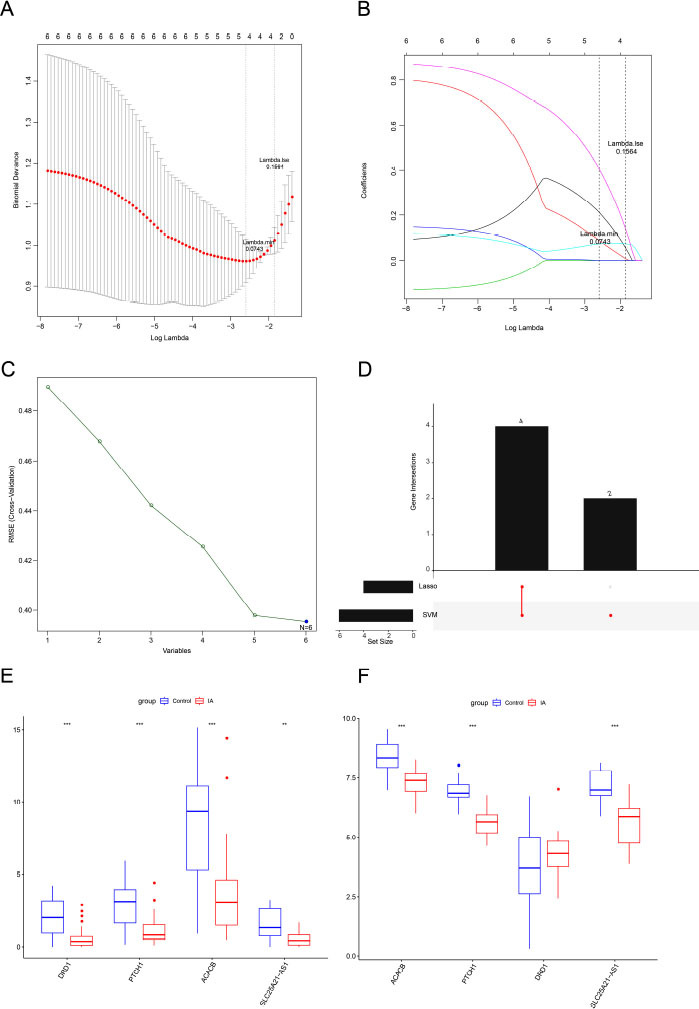
Identification of final hub genes in IA by LASSO and SVM-RFE analyses. (**A**) The plot of LASSO penalty term parameters with log (lambda) values in horizontal coordinates and degrees of freedom in vertical coordinates. (**B**) LASSO regression coefficients plot with log (lambda) in the horizontal coordinates and the coefficients of the genes in the vertical coordinates, demonstrating the changes in the coefficients of different variables with the λ penalty. (**C**) The plot of SVM-RFE generalized error versus several features, with the horizontal coordinate being the number of genes included within the model at each iteration and the vertical coordinate being the root mean square error. (**D**) The plot of LASSO versus SVM-RFE to obtain intersection upsets of special feature genes. (**E**) Boxplot of biomarker expression in the training set of the GSE122897 dataset (44 IA tissue samples and 16 intracranial cortical artery control samples). (**F**) Box line plot of biomarker expression in the GSE75436 test set (15 IA tissue samples and 15 temporal superficial artery control samples). ns represents *p* > 0.05; *p < 0.05, **p < 0.01, ***p < 0.001, and ****p < 0.0001.

We selected four feature genes based on the LASSO algorithm and lambda.min=0.0743 (Figs. **5A-B**). Simultaneously, we performed SVM-RFE analysis on the six intersecting genes using the R package “e1071”. The model achieved optimal performance with all six genes, resulting in minimal error (Fig. **5C**). *DRD1*, *PTCH1*, *ACACB,* and *SLC25A21*-*AS1* were the common genes identified by the two machine learning algorithms (Fig. **5D**). The prognostic performance of the four genes was tested using the GSE75436 cohort (the training set). While IA samples in the GSE122897 training cohort had lower *DRD1* gene expression than the control samples (Figs. **5E** and **F**), there was no significant difference in the GSE75436 training group. In addition, as *PTCH1*, *ACACB,* and *SLC25A21*-*AS1* showed significantly lower expression in the IA samples in both the training and test sets (*p*<0.05), these three genes were considered as the key genes in subsequent studies.

### 3.6 Biomarkers correlated with Immune Infiltration in IA

**Fig. (6):**
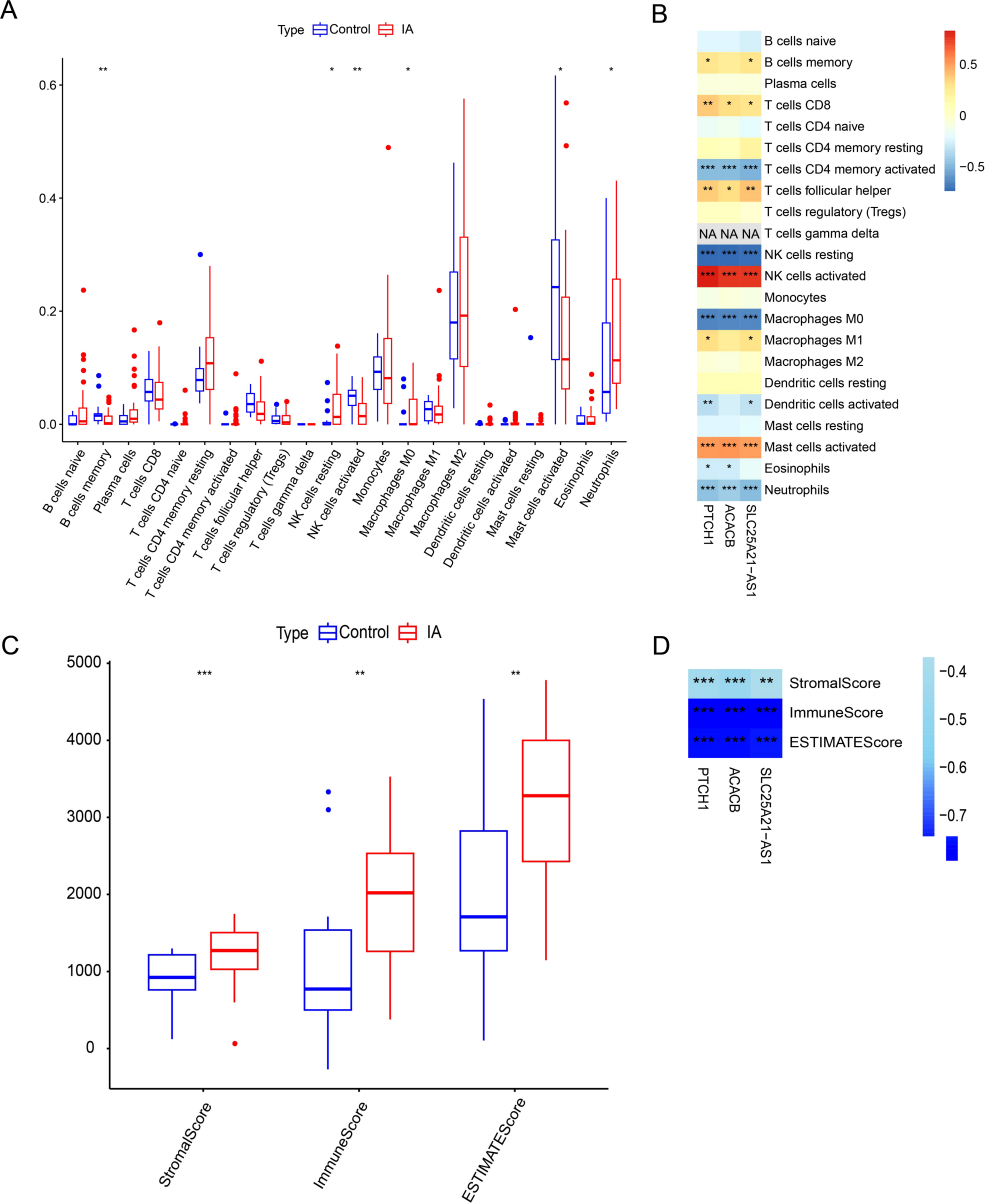
Correlation analysis of 3 biomarkers (*PTCH1*, *ACACB*, and *SLC25A21-AS1*) with the degree of immune cell infiltration. (**A**) Box line plot of immune cell infiltration in 22 cell types as estimated by CIBERSORT. (**B**) Spearman correlation analysis of 3 biomarkers with immune infiltration status. (**C**) Box line plot of the immune infiltration profile of ESTIMATE. (**D**) Spearman correlation analysis of 3 biomarkers with immune infiltration status. ns represents *p* > 0.05; *p < 0.05, **p < 0.01, ***p < 0.001, and ****p < 0.0001.

Using the “CIBERSORT” package, we compared immune cell infiltration patterns between IA and control samples in the training set. IA samples exhibited significantly higher immune infiltration of memory B cells, resting NK cells, and infiltrating neutrophil cells than control samples (Fig. **6A**, *p*<0.05), while that of macrophages M0 and activated mast cells was considerably lower than NK cells in IA samples (Fig. **6A**, *p*<0.05). Next, correlation analysis between immune cell infiltration scores and the three genes showed that the expressions of *PTCH1*, *ACACB,* and *SLC25A21*-AS1 were negatively related to the infiltration of T cells, CD4 memory activation, resting NK cells, macrophages M0, and neutrophils (Fig. **6B**). Conversely, these three genes were positively related to the infiltration of CD8 T cells, follicular helper T cells, activated NK cells, and activated mast cells. IA samples showed notably higher ESTIMATEScore, ImmuneScore, and StromalScore than the control samples (Fig. **6C**). These three scores were negatively linked to the expression of the three genes (*p*<0.01, Fig. **6D**). The above findings indicated that immune cell infiltration in IA may be intimately linked to the three genes.

### 3.7 Development of a TF-miRNA Regulatory Network

Potential TFs targeting the key genes were predicted using the “TFBSTools” package and the JASPAR database. As shown in Fig. (**7**) analysis revealed 67, 71, and 76 potential regulatory TFs targeting *PTCH1*, *ACACB,* and *SLC25A21*-*AS1*, respectively. For miRNA prediction, we identified 9 experimentally validated miRNAs targeting *PTCH1*, and each miRNA was validated by at least 5 experiments.

## 4 DISCUSSION

IA rupture is a critical cerebrovascular event that is frequently undetected until rupture due to its clinically asymptomatic progression. However, current strategies for rupture risk prediction remain limited [4], underscoring the need to identify reliable biomarkers and therapeutic targets. Compared with the previous study applying WGCNA and immune infiltration analysis to neurovascular diseases, the present study has involved several innovative aspects. For example, previous research has combined differential analysis with machine learning to screen key genes related to oxidative stress in ischemic stroke patients, and found these genes to be involved in reactive oxygen species metabolism, lipid deposition, and cell migration, suggesting that they may play a role in the occurrence and development of stroke [37]. Another study integrated WGCNA with three machine learning algorithms to link polycystic ovary syndrome with atherosclerosis and identified *MMP9* and *P2RY13* as potential biomarkers. Additionally, immune infiltration analysis revealed an abnormal distribution of monocyte and macrophage subpopulations in the pathological process of atherosclerosis. Ultimately, a diagnostic model was established to assess the risk of polycystic ovary syndrome-related atherosclerosis [38]. Different from previous studies, this study was the first to combine WGCNA with two machine learning algorithms (Lasso and SVM-RFE) with a focus on aging and inflammation as entry points to systematically screen key genes closely associated with IA and further explore their correlation with immune cell infiltration. While earlier studies have primarily emphasized oxidative stress or metabolic abnormalities, this study has highlighted the interactive relationship among aging, inflammation, and immunity, thereby broadening our understanding of the mechanisms underlying the development of IA and providing novel molecular insights for its early diagnosis and targeted intervention.

Our integrated analysis identified *PTCH1*, *ACACB,* and *SLC25A21*-*AS1* as key genes with downregulated expression in the IA samples in both the training and test sets. Existing evidence supports their involvement in various pathological contexts. For example, *PTCH1* promoted the progression of medulloblastoma by preventing oncogene-induced cellular senescence [39], while its inhibition suppressed the levels of systemic inflammatory proteins in mice [40]. This indicated a dual role of *PTCH1* in regulating senescence and inflammation. *ACACB*, known for its metabolic functions, can upregulate pro-inflammatory cytokines in renal epithelial cells [41] and is also associated with drug resistance in colorectal cancer through modulation of EGFR signaling [42]. Similarly, long non-coding RNA *SLC25A21-AS1* has been found to inhibit the proliferation and metastasis of ovarian cancer cells, showing a tumor-suppressive role in ovarian cancer [43, 44]. These findings have indicated critical roles of these genes related to cellular senescence and inflammation in IA pathogenesis. Cellular senescence can trigger senescence-associated secretory phenotype (SASP), which is characterized by the release of pro-inflammatory cytokines and chemokines that amplify inflammatory signaling within the vascular microenvironment [45, 46]. In turn, chronic inflammation may reinforce or sustain the senescent state in vascular cells, forming a feed-forward loop that promotes extracellular matrix degradation, endothelial dysfunction, and vessel wall instability, which are hallmark features of IA development [6, 47, 48]. Therefore, the downregulation of *PTCH1*, *ACACB*, and *SLC25A21-AS1* may disrupt this regulatory balance, exacerbating both senescence-associated inflammation and immune dysregulation, ultimately contributing to IA progression. Nevertheless, further mechanistic studies are needed to validate these hypotheses and confirm their specific roles in the senescence-inflammation axis of IA.

The TME is a dynamic ecosystem characterized by complex interactions among tumor cells, stromal cells, endothelial cells, and both innate and adaptive immune components [49]. T follicular helper (Tfh) cells enhance anti-tumor responses by activating B cells [50]. Quiescent NK cells can promote tumor dissemination through tumor-derived stem cell factors [51], whereas activated NK cells exert cytotoxic effects on the inflammatory environment [52]. Memory B cells possess high plasticity and heterogeneity, and their prolonged survival and rapid activation may contribute to malignant transformation in various B-cell-related cancers [53]. Additionally, interactions between M0 macrophages and naive CD4+ T cells could facilitate the formation of an immunosuppressive microenvironment in cervical cancer [54]. Mast cells, depending on their state and context, can either enhance or suppress tumor progression *via* cytokine secretion and interaction with immune and stromal cells [55]. CD8+ T cells are widely recognized as key anti-tumor effectors and serve as biomarkers for immunotherapy efficacy [56]. In our study, we observed that the abundance of memory B cells, resting NK cells, and neutrophils was significantly higher in IA samples compared to controls, while that of activated NK cells, M0 macrophages, and activated mast cells was reduced. Furthermore, the expression of *PTCH1*, *ACACB*, and *SLC25A21-AS1* was negatively correlated with the infiltration of CD4 memory T cells, resting NK cells, M0 macrophages, and neutrophils, but positively correlated with CD8+ T cells, Tfh cells, activated NK cells, and activated mast cells. These correlations suggested that downregulation of the three genes may contribute to an immunosuppressive or immune-dysregulated environment in IA. For example, lower infiltration of CD8+ T cells and activated NK cells, which are essential for cytotoxic immune responses, may weaken immune surveillance and contribute to disease progression. Conversely, higher infiltration of neutrophils and M0 macrophages is often associated with tissue damage and chronic inflammation, which can promote vascular remodeling and destabilization [57, 58]. These key processes are well-established key mechanisms in IA pathology [59]. Importantly, the above findings indicated that *PTCH1*, *ACACB*, and *SLC25A21-AS1* may regulate immune infiltration through pathways related to aging and inflammation in IA. The differential expressions of these genes could serve as potential modulators of the immune microenvironment and diagnostic or prognostic biomarkers for IA. Hence, understanding these regulatory relationships could offer new therapeutic strategies to restore immune balance in IA patients through targeting key immune cell populations or reactivating senescence-cleared immune responses.

The present research study has involved several limitations. First, this study was primarily based on two publicly available GEO database datasets, which had a small sample size and potential influences from data heterogeneity and batch effects that may have affected model stability and the generalizability of conclusions. Subsequent studies will combine more independent cohorts (including clinically collected samples) for cross-validation, expand the sample size, and use batch correction algorithms to improve the robustness and applicability of the results. Secondly, our results relied entirely on transcriptome data from public databases and computer simulation analyses. Although computational analyses and machine learning algorithms can mine potential key genes and reveal potential mechanisms in large-scale data, these predictive findings lack direct experimental validation. Future research should systematically explore the functions of these genes using both *in vitro* and *in vivo* experiments. Genetic interventions in vascular endothelial cells and smooth muscle cells could be employed to examine their effects on cellular senescence markers, inflammatory factor secretion, and functional status. Furthermore, such studies should examine the regulatory roles of these genes in vascular structural changes and inflammatory infiltration using relevant IA animal models. Additionally, techniques, such as immunohistochemistry, flow cytometry, and single-cell sequencing, could be employed to validate the association between these genes and specific immune cell subsets. To further elucidate their regulatory mechanisms, chromatin immunoprecipitation quantitative PCR (ChIP-qPCR) and dual-luciferase reporter assays may be employed to verify interactions with predicted TFs and miRNAs. Through this progressive experimental framework, we can validate the predictive results of this study and further clarify the specific role of the aging-inflammation-immunity axis in IA, thereby providing a more solid theoretical and experimental basis for their early diagnosis and targeted treatment. Third, although we constructed a TF-miRNA regulatory network for the key genes, these interactions require experimental validation. Future studies are encouraged to analyze the regulatory relationship between the TFs and key genes and their role in the senescence-inflammation-immune regulation axis through transcription factor chip analysis, luciferase reporter gene experiments, and other related methods. Finally, our stringent intersectional approach for selecting common genes from both training and validation datasets excluded potentially relevant candidate *DRD1*, which might play an important role in a particular dataset. To overcome this limitation, we plan to introduce weighted scoring or ensemble modeling strategies that allow for a certain degree of data variability to expand candidate gene coverage and establish a priority-ranked pool of targets for subsequent functional characterization.

## CONCLUSION

This study utilized data from public databases in combination with WGCNA and two machine learning algorithms to identify *PTCH1*, *ACACB*, and *SLC25A21-AS1* as three potential biomarker genes associated with senescence and inflammation for IA. The results showed that the three genes may be closely related to changes in the IA immune microenvironment. This study has provided novel insights into the molecular pathogenesis of IA and laid a foundation for further exploration of their potential role in immune regulation and early diagnosis. However, further experimental validation is needed to clarify their functional and clinical application value in IA.

## Figures and Tables

**Fig. (7):**
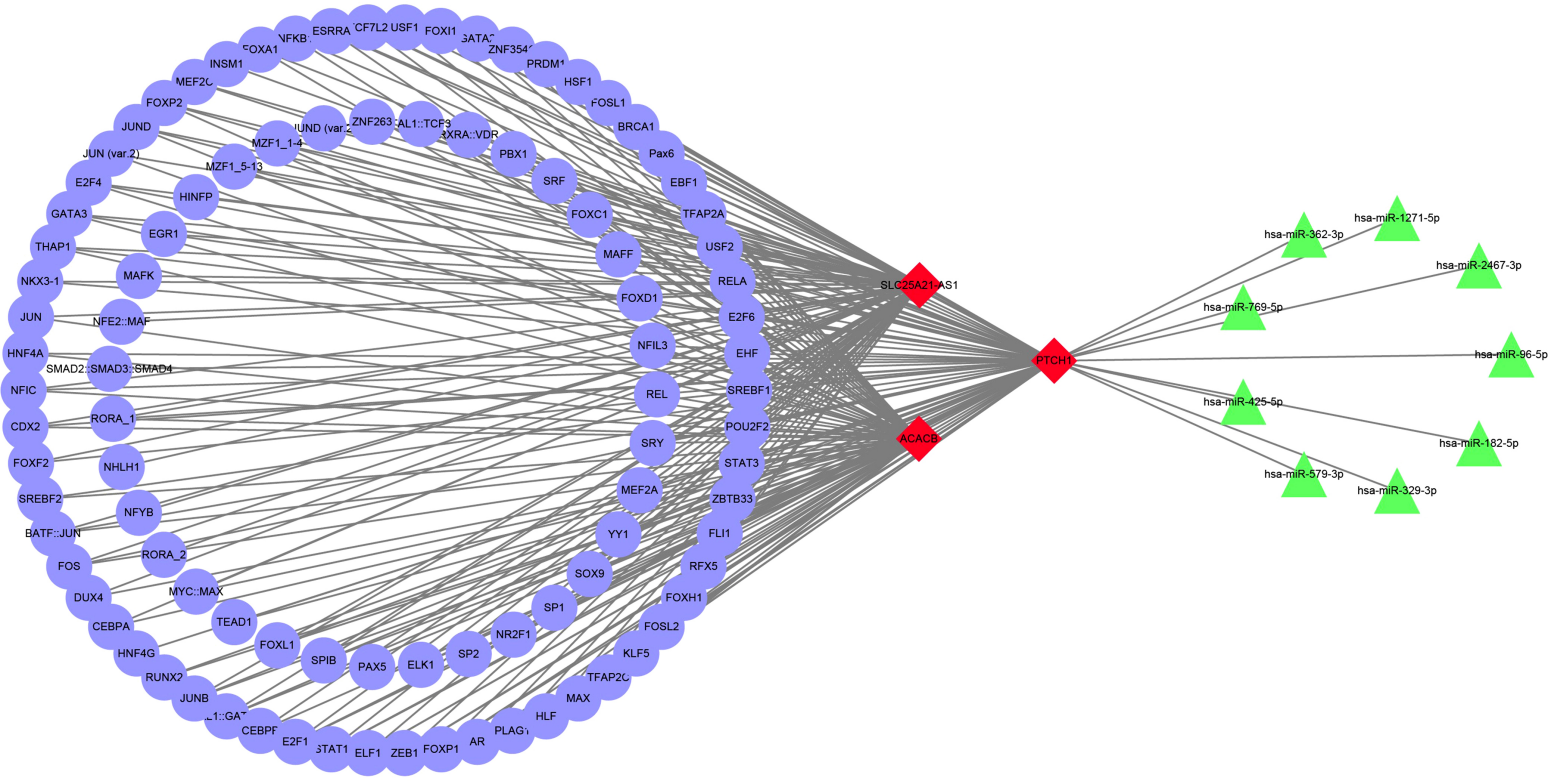
TF-miRNA regulatory network. Red diamonds represent biomarkers, blue circles represent TF transcription factors, and green triangles represent miRNAs.

## Data Availability

The datasets generated and/or analyzed during the current study are available in the GSE122897 repository (https://www.ncbi.nlm.nih.gov/geo/query/acc.cgi?acc=GSE122897) and GSE75436 repository (https://www.ncbi.nlm.nih.gov/geo/query/acc.cgi?acc=GSE75436).

## LIST OF ABBREVIATIONS

IA: Intracranial Aneurysm
WGCNA: Weighted Gene Co-Expression Network Analysis
SVM-RFE: Support Vector Machine Recursive Feature Elimination
LASSO: Least Absolute Shrinkage and Selection Operator
GEO: Gene Expression Omnibus
ESTIMATE: Estimation of Stromal and Immune Cell Expression in Malignant Tissues
DSigDB: Drug Signatures Database
Cellage: Cell Senescence
GS: Gene Significance
MM: Module Membership
DEGs: Differentially Expressed Genes
CC: Cellular Component
MF: Molecular Function
BP: Biological Process
GO: Gene Ontology
KEGG: Kyoto Encyclopedia of Genes and Genomes
GSVA: Gene Set Variant Analysis
ssGSEA: Single-Sample Gene Set Enrichment Analysis
TF: Transcription Factor
