# Supplementary material for: Identification of Senescence- and Inflammation-Related Genes and Immune Microenvironment Characterization in Intracranial Aneurysms
Source: Endocr Metab Immune Disord Drug Targets. 2026 Jan 2;26:E18715303420035. doi: 10.2174/0118715303420035251023051847 (PMC13334262; doi:10.2174/0118715303420035251023051847)
Supplement: Supplementary file 1 [file EMIDDT-26-E18715303420035_SD1.pdf]

Supplementary Material

Identification of Senescence- and InflammationRelated Genes and Immune Microenvironment Characterization in Intracranial Aneurysms

Xiaoyan Li[1], Yingying Li[2], Le Zhang[1], Jie Mao[3,*] and Bin Li[1,*]

[1]*Department of Neurology, Anhui No.2 Provincial People's Hospital, Hefei, 230041, China;* [2]*Graduate School, Bengbu Medical University, Bengbu, 233000, China;* [3]*Department of Neurosurgery, Longgang Central Hospital of Shenzhen, Shenzhen, 518035, China*

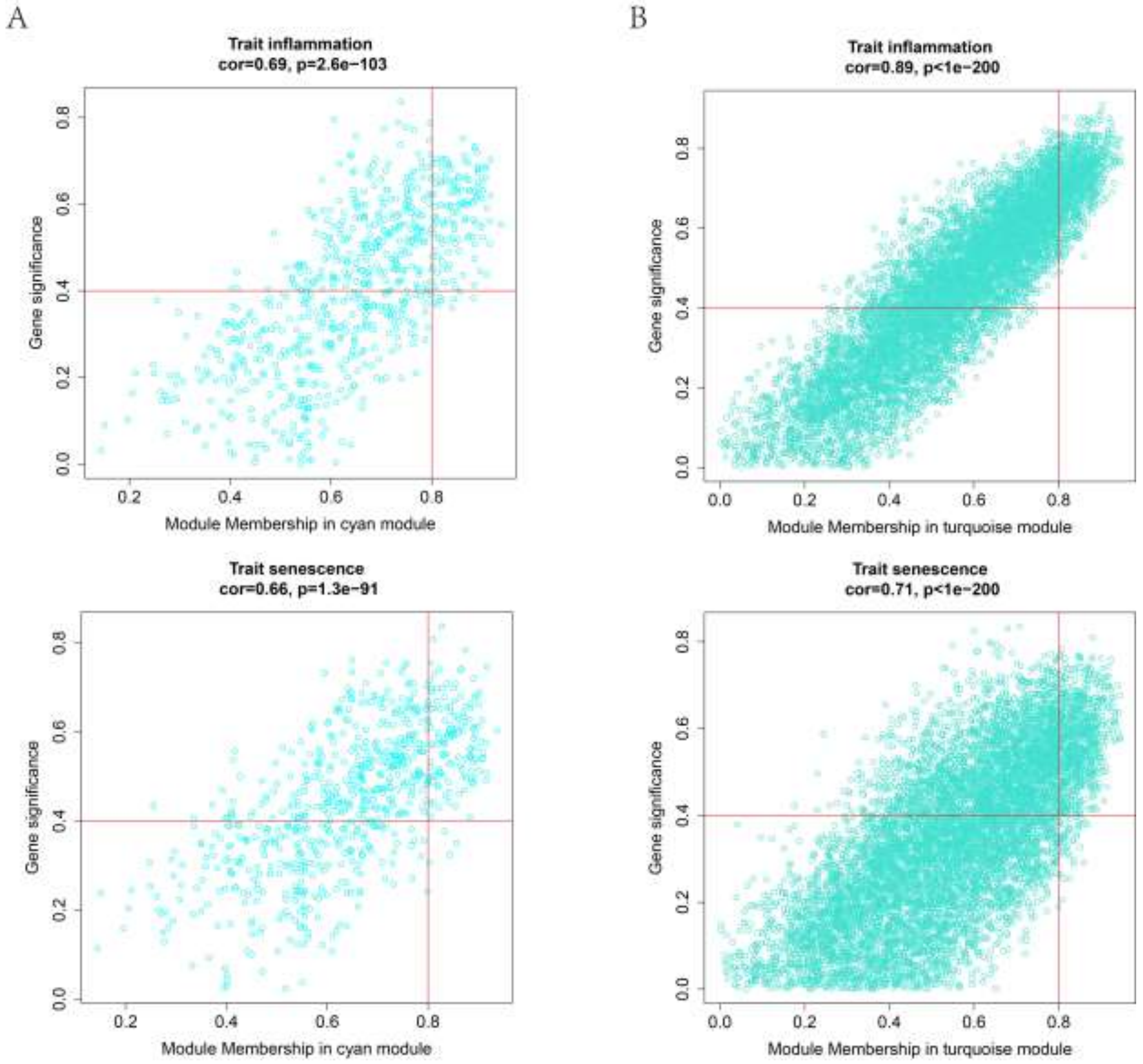

Figure S1. Analysis of the correlation between module gene significance and inflammatory and aging traits. (**A**) GS-MM plot of the MEcyan module, with MM, the correlation of each gene with the trait gene, as the horizontal coordinate. The vertical coordinate is GS, which is the correlation between genes and traits within the module. (**B**) GS-MM plot of the MEturquoise module.
